# The Concept and Components of Engagement in Different Domains Applied to eHealth: A Systematic Scoping Review

**DOI:** 10.3389/fpsyg.2020.00926

**Published:** 2020-05-27

**Authors:** Saskia M. Kelders, Llewellyn Ellardus van Zyl, Geke D. S. Ludden

**Affiliations:** ^1^Department of Psychology, Health and Technology, Centre for eHealth and Wellbeing Research, University of Twente, Enschede, Netherlands; ^2^Optentia Research Focus Area, North-West University, Vanderbijlpark, South Africa; ^3^Department of Industrial Engineering, University of Eindhoven, Eindhoven, Netherlands; ^4^Department of Human Resource Management, University of Twente, Enschede, Netherlands; ^5^Institut für Psychologie, Goethe University, Frankfurt, Germany; ^6^Department of Design, Production and Management, University of Twente, Enschede, Netherlands

**Keywords:** engagement, eHealth, mHealth, systematic scoping review, positive organizational e-interventions

## Abstract

Within the context of eHealth interventions, a shared understanding of what constitutes engagement in and with eHealth technologies is missing. A clearer understanding of engagement could provide a valuable starting point for guidelines relating to the design and development of eHealth technologies. Given the cross-disciplinary use of the term “engagement,” investigating how engagement (and its components) is conceptualized in different domains could lead to determining common components that are deemed important for eHealth technological design. As such, the aim of this paper was 3-fold: (a) to investigate in which domains engagement features, (b) to determine what constitutes engagement in these different domains, and (c) to determine whether there are any common components that seem to be important. A comprehensive systematic scoping review of the existing literature was conducted in order to identify the domains in which engagement is used, to extract the associated definitions of engagement, and to identify the dimensionality or components thereof. A search of five bibliographic databases yielded 1,231 unique records. All titles, abstracts, and full texts were screened based on specific inclusion and exclusion criteria. This led to 69 articles being included for further analyses. The results showed that engagement is used in seven functional domains, categorized as follows: student (*n* = 18), customer (*n* = 12), health (*n* = 11), society (*n* = 10), work (*n* = 9), digital (*n* = 8), and transdisciplinary (*n* = 1) domains. It seems that some domains are more mature regarding their conceptualization and theorizing on engagement than others. Further, engagement was found to be predominantly conceptualized as a multidimensional construct with three common components (behavior, cognition, and affective) shared between domains. Although engagement is prolifically used in different disciplines, it is evident that little shared consensus as to its conceptualization within and between domains exists. Despite this, engagement is foremost seen as a state of being engaged in/with something, which is part of, but should not be confused with, the process of engagement. Behavior, cognition, and affect are important components of engagement and should be specified for each new context.

## Introduction

Developing scalable, cost-effective, and efficient technological solutions to enhance the general health and well-being of individuals has become vital within today's digital economy (Stander and van Zyl, 2019). Positive organizational interventions that focus on harnessing and improving individuals' strengths to increase employees' well-being, and organizational outcomes are examples that have begun to gain more attention (Winslow et al., 2017; Salanova and Ortega-Maldonado, 2019). Designing these types of solutions requires designers to ensure that technological interventions (such as health apps and web-based platforms) not only are effective and usable but also have the potential to actively immerse consumers and users in its content (Couper et al., 2010). If individuals are able to actively engage with such technologically driven interventions, they could potentially reap all the associated physical and psychological health benefits that it may bring. However, it has been shown and argued that technologically driven interventions often do not fully engage people, thereby limiting the effectiveness thereof (Christensen et al., 2009; Donkin et al., 2011; Kelders et al., 2012; Perski et al., 2017). Designing engaging technological interventions is therefore a crucial success factor to consider. Although there is considerable agreement in the literature in support of this argument, as well as the benefits that engagement yields, little consensus exits with regard to what engagement is and how it should be conceptualized.

In eHealth, the use of technology to support health and well-being, a much-documented issue related to a lack of engagement, is *non-adherence*. Often, people who use an eHealth solution do not use the offered technology the way in which the developers intended; this is what researchers refer to as non-adherence (Christensen et al., 2009; Kelders et al., 2012). Examples are participants not completing all lessons within an eMental health intervention, or not using all of the functions within a diabetes management system. Research has shown that there is a dose—response relationship: for people who use a technology more, the positive effects are greater (Donkin et al., 2011; Yeager et al., 2018). However, not all eHealth interventions show this relationship, and it has been argued that this has to do with the way adherence is conceptualized. The assumption that increased frequency of use equates to “better results” does not necessarily ring true (Sieverink et al., 2017; Kelders, 2019). Also, it seems that the reasons why people choose to use an intervention might be more important than the frequency or duration of its use. Research shows that when users feel involved in, or are able to identify with the intervention, the effects may be larger (Donkin and Glozier, 2012; Kelders, 2015). Similarly, a review on engagement in digital health interventions described engagement as the extent of usage and a subjective experience characterized by attention, interest, and affect (Perski et al., 2017). This definition clearly describes engagement to be more than only usage of a system. However, the majority of articles included in that review only viewed engagement in behavioral terms, that is, as usage. This call to see and measure engagement not (just) through usage data is shared by more researchers (Yardley et al., 2016; Short et al., 2018) not only within the field of eHealth technologies (O'Brien and Toms, 2008; Doherty and Doherty, 2018).

It is important to note here that both the content and the design (the way the content is delivered) of the intervention may influence users' level of engagement or adherence. The design of a technological solution, its aesthetics, functionality, and behavior, is an important precursor to individuals' engagement, because such actively influences their experience of—their emotional connection to—and the behavior directed toward the intervention (Desmet and Hekkert, 2007; Ludden et al., 2012; Niedderer et al., 2017). When controlling for intervention content, providing users with a highly immersive, personalized intervention experience seems to be more effective in enhancing outcomes than providing them with static, linear, and unengaging content (Couper et al., 2010; Kelders et al., 2018). In other words, the design of a technologically driven intervention strongly influences how it is perceived, how it is employed, and how effective it will eventually be (Ludden et al., 2015; Kelders, 2019). Given the importance that the design of a technological solution poses for both engagement and adherence, it is not surprising that it has become a centrally debated topic within the domains of Interaction Design and Human Computer Interaction in recent years (Doherty and Doherty, 2018). Researchers from these fields actively advocate for the design of highly engaging and immersive user experiences in order to enhance utilization and manage non-adherence. However, despite its relative importance, there is still no generally accepted model or theory on how design influences engagement. Overbeeke and colleagues (Overbeeke et al., 2004), for example, stated that engagement in interaction should be reinstated by a focus on the physicality of the product. In their line of reasoning, the aesthetics of interaction (the sensory pleasure that people experience through interaction) play an important role in engrossing individuals within the interaction. In contrast, Gulotta and colleagues (Gulotta et al., 2016) argued that the active use of a technologically driven intervention is a function of an alignment between an individual's desire for and ability to achieve a specific outcome with said system. Here, the alignment between the personal characteristics of the user and the nature of the design seems to be an important factor for engagement. These types of inconsistencies in the literature result in confusion as to how intervention platforms should be designed in order to enhance engagement.

It is therefore clear that despite the agreement among all disciplines from which eHealth intervention research draws as to the importance of engagement, a commonly shared conceptualization of such is lacking. As engagement is a broad concept that has been used in many domains, it seems useful to look at how other domains define and use engagement in order to capitalize on such within eHealth intervention design. Whereas in eHealth the discussion on what engagement constitutes is just emerging, other domains have a rich tradition in studying engagement [e.g., patient engagement (Carman et al., 2013) and work engagement (Bakker et al., 2008)]. Important discussions in these domains are, for example, whether engagement should be seen as a process (getting and remaining engaged and/or disengaged) or a state (of being engaged) (Sonnentag, 2017). In these domains, engagement is seen as a multidimensional construct consisting of multiple components (Graffigna, 2017), mirroring trends within eHealth research that engagement is more than just “usage.” Insight in what these components are in other domains might be a particularly timely step forward to better understand engagement in eHealth technologies. A better understanding of engagement in and with eHealth technologies can provide a much necessary starting point for guidelines for the design and development of eHealth technology.

As such, the aim of this systematic scoping review is to gain a better understanding of in what domains the concept of engagement features and what constitutes engagement in these different domains and to determine whether there are any common components that seem to be important. This systematic scoping review will focus on all domains where engagement is used as a concept, providing that engagement means something more than only using or doing something (e.g., engaging in warfare). Focus will be on which components of engagement are commonly identified and how such can be translated into eHealth intervention research.

## Methods

### Research Approach

In order to determine how engagement is conceptualized, and to determine the global factors underpinning such, a systematic scoping review was conducted in line with existing guidelines (Peters et al., 2015). A systematic scoping review was deemed the appropriate method because of its focus on mapping the concept of engagement (Arksey and O'Malley, 2005). This approach is particularly useful to synthesize and summarize knowledge about an objective in question that exhibits a high level of heterogeneity and complexity that spans disciplines (Horsley, 2019).

### Search Strategy

**Figure 1:**
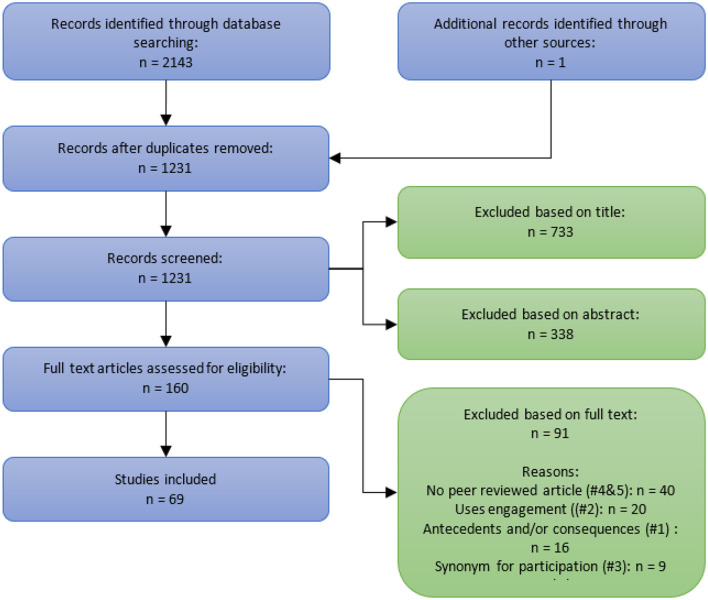
Flowchart of article selection.

A comprehensive, systematic literature search was conducted between August 2018 and January 2019 in the following bibliographic databases: Scopus, Web of Science, Science Direct, PsycINFO, and ACM Digital Library. The databases were queried with a combination of the terms “engagement” AND “concept OR theory OR definition” occurring in the title of published, peer-reviewed articles. The last run was conducted on 18 January 2019. With the use of this search string, 2,143 titles were identified from 1994 up until 2018 (see Figure 1 for the flow diagram of article selection).

### Eligibility Criteria

This review aimed to identify peer-reviewed academic articles (seminal works) that aimed to provide a definition, conceptualization, or theory of engagement, within any discipline. Only academic peer-reviewed scientific papers and conference proceedings that were published in English were eligible for inclusion in this study. Exclusion criteria were as follows:

1. Papers that only focused on antecedents or consequences of engagement and did not include a focus on the concept of engagement itself, for example, papers that solely aimed to explain or predict engagement (e.g., trust and usability) or focus on factors that resulted from engagement (e.g., enhanced performance), which did not specifically focus on conceptualizing engagement itself. This exclusion criterion was deemed relevant because of the large number of studies that focus solely on antecedents and consequences but do not provide any (new) information on the concept of engagement itself. Studies that state that they (also) focus on the concept of engagement were not excluded.
2. Papers that solely used the concept of engagement as a metric or as part of a larger empirical model, for example, papers aimed at using engagement as a factor in a structural model. This exclusion criterion was deemed relevant because of the large number of studies that only used a measure of engagement as part of a larger empirical model, while not providing additional information on the concept of engagement itself.
3. Papers that only employed engagement as a synonym for another term or to indicate action (e.g., interaction with the press and engagement in warfare).
4. Unpublished masters or doctoral theses.
5. Textbooks and book chapters were also excluded, because many textbooks and book chapters provide more of an overview of earlier work than new insights as original research papers do, and not all textbooks and book chapters are peer reviewed.

### Study Selection

Study selection was done in two steps. First, after duplicates were removed, the titles and abstracts of all retrieved articles were screened for eligibility by two authors (SK and LvZ or GL). Next, the full text of all remaining publications was checked for inclusion by two authors (SK and LvZ or GL). Disagreements on the inclusion or exclusion of publications were discussed until agreement was reached. The average percentage of agreement between authors was approximately 90%, which is higher than the suggested 70% overlap (Booth et al., 2012). To check whether seminal works had been overlooked during the initial search process, included papers were checked whether they referred to any important publications that were not yet included.

### Data Extraction

The characteristics of all included studies were extracted by one author (SK). Data extraction of 20% of the included studies (*n* = 14) was validated by the other authors. Data items that were extracted from each included study were country of origin, year of publication, type and subtype of engagement, purpose of the study, used methods, and main findings. Furthermore, for each included paper, the definition of engagement used was extracted. Here, it is was indicated whether this was a process definition (i.e., a definition about the process of getting and remaining engaged and/or disengaged); whether the definition was newly developed or already existing; or if no specific definition was chosen or if the definition was unclear. Lastly, whether engagement was said to consist of multiple components, and what these components were, was extracted.

## Results

### Study Selection

The search yielded 1,231 unique titles after duplicates were removed. After title, abstract, and full text screening, 69 articles were included (Figure 1). In total, 91 titles were excluded based on the full text. The most common reason for exclusion was that the titles were not peer-reviewed articles (*n* = 40). Of these, many were book chapters that are often not peer-reviewed and/or provide more of a summary of earlier work than new studies. Twenty titles were excluded because they included the concept of engagement in the title, but the study was not concerned with engagement itself and therefore did not provide any new insights on the concept or definition. Another 16 articles were excluded because they focused on antecedents or consequences of engagement, but not on the concept itself. Although these papers are interesting, they were excluded from this review because the focus is on what constitutes engagement and not on antecedents or consequences. Nine publications were excluded because in the full texts it became clear that engagement was not used as a concept in itself but only as a synonym for participation, involvement, or to action something specific. Lastly, six studies were excluded because the full texts were not in English.

### Characteristics of Included Studies

In total, 69 papers published from 1990 up until 2018 were included. Publications were sparse from 1990 until 2007 (a total of *n* = 7); however, it increased substantially afterwards, with a peak in 2017 (*n* = 14). Almost half of the publications emanated from the USA (*n* = 31), 21 publications originated from Europe, 10 from Australia and New Zealand, and three from Canada and the others ranging from United Arab Emirates to Japan. We categorized the papers into seven domains of engagement: student (*n* = 18), customer (*n* = 12), health (*n* = 11), society (*n* = 10), work (*n* = 9), digital (*n* = 8), and transdisciplinary (*n* = 1). Each category is discussed below. For each category, the characteristics of the studies are provided in a separate table. All definitions used in the different studies are provided in Appendix 1.

#### Student Engagement

Table 1 shows the characteristics of the 18 studies classified as student engagement. Of these studies, 11 used the term student engagement, and three focused on school engagement, two on agentic engagement (as a component of student engagement), one on reading engagement, and one on academic engagement. Eleven studies analyzed the concept of engagement by discussing or reviewing literature. Four studies focused on developing and testing a scale to measure engagement, using both qualitative and quantitative methods. Three studies used qualitative methods to investigate a stakeholder perspective of the concept and its components. Eight studies used one or more existing definitions of engagement for their study and aimed to get more insight in this definition, whereas seven studies resulted in a new definition. In three studies, it was unclear what definition of engagement the authors have used.

**Table 1:** Characteristics and findings of student engagement studies.

**Study and subtype**	**Purpose**	**Method**	**Main findings**	**Definition and components**
(Appleton et al., 2008); student	Analyze concept	Discuss literature	Need for consensus and clarity	None chosen; behavioral, affective/emotional, psychological, cognitive, academic
(Barkaoui et al., 2015); student	Stakeholder perspective of concept	Qual.	Need for contextualization, antecedents explored	None chosen
(Bernard, 2015); student	Analyze concept	Systematic review	Lack of clarity and consensus	New (process); behavioral, cognitive, emotional
(Burch et al., 2015); student	Propose concept + test scale	Quant.	Model of scale confirmed	New; emotional, physical, cognitive in class, cognitive out of class
(Ciric and Jovanovic, 2016); student	Analyze concept	Discuss literature	Concept is dynamic, malleable, multidimensional, and interrelated	Existing; emotional, cognitive, behavioral
(Fredricks et al., 2004); school	Analyze concept	Discuss literature	Richer characterizations of components are needed	Existing; behavioral, emotional, cognitive
(Harris, 2008); student	Stakeholder perspective of concept	Qual.	Six different ways of understanding student engagement were found	Unclear; behavioral, psychological, cognitive
(Harris, 2011); student	Stakeholder perspective of concept	Qual.	Six different ways of understanding student engagement were found + three ways of facilitating engagement	Unclear; behavioral, psychological, cognitive
(Hollingshead et al., 2018); student	Stakeholder perspective of (components of) concept	Qual.	Importance of and insight in components for specific target group	Existing; behavior, cognition, affect
(Jimerson et al., 2003); school	Analyze concept + measures	Systematic review	Many terms and measurements used; items classified in contexts	New; affective, behavioral, cognitive
(Lawson and Lawson, 2013); student	Analyze concept	Discuss literature	New definition as a system of constructs and a process	New (process)
(Liem and Martin, 2012); student	Describe and discuss concept + measurement	Discuss literature	Scale is a meaningful contribution to research and practice	Existing; adaptive cognition, adaptive behavior, maladaptive cognition, maladaptive behavior
(Montenegro, 2017); agentic	Analyze concept	Discuss literature	Agentic engagement is a consistent researchable field	Existing; agentic, (behavior, cognition, emotion)
(Reeve, 2013); agentic	Introduce concept + measurement	Quant.	Agentic engagement scale was developed and tested	Existing; agentic, (behavior, cognition, emotion)
(Schuetz, 2008); student	Develop and test new conceptual model	Qual. + quant.	Model and scale confirmed	Existing; interest, mindfulness, cognitive effort, deep processing of new information
(Skinner et al., 2009); academic	New conceptualization	Discuss literature + quant.	Scale developed and tested	New; behavioral, emotional
(Unrau and Quirk, 2014); reading	Analyze concept	Discuss literature	Concept is often blurred; constructs clarified	New; affective, individual participation, cognitive
(Wang et al., 2019); school	Analyze concept + develop and validate scale	Discuss literature + qual. + quant.	Scale developed and tested; aspects confirmed	New; behavioral, emotional, cognitive

Looking at the components of engagement in this category, there seems to be some consensus: 11 studies mentioned behavior (or physical), affect (or emotion), and cognition as components of engagement. However, within these studies, there is much discussion on what exactly these components entail. Another five studies mention two of these components, or suggest other components besides behavior, affect and cognition, for example, agentic engagement. The last study also identifies cognition (cognitive effort) and adds other components as interest and mindfulness. Furthermore, two studies explicitly mention that the opposite of engagement (termed disengagement or disaffection) is also a construct that needs to be conceptualized (Skinner et al., 2009; Wang et al., 2019). Lastly, multiple authors stress the need for each study to be clear about what conceptualization of engagement is used (Jimerson et al., 2003; Appleton et al., 2008; Unrau and Quirk, 2014; Bernard, 2015).

#### Customer Engagement

Table 2 shows the 12 studies classified as *customer engagement*. These studies used variations of the term for their specific form of engagement (customer or consumer; with or without “brand”) but often did not differentiate between the terms. Interestingly, almost all studies yielded a new definition of engagement. Of the two that did not, one was a follow-up from an earlier study of the same author to evaluate the conceptualization of the previous study, and the other developed a new typology and model but was unclear about the specific definition. Four studies analyzed and applied the concept of consumer engagement to a new area, for example, sports fans, mobile technology, and health care. Eight studies included qualitative or quantitative data and the same number of studies reviewed literature.

**Table 2:** Characteristics and findings of customer engagement studies.

**Study and subtype**	**Purpose**	**Method**	**Main findings**	**Definition and components**
(Abdul-Ghani et al., 2018); consumer	Apply to specific area + stakeholder perspective	Qual.	Conceptual framework with engagement cycle in C2C contexts	New; cognitive, affective, self-image, motivation
(Bowden, 2014); customer	Analyze concept	Discuss literature	Conceptual framework with antecedents and consequences	New (process)
(Brodie et al., 2011); customer	Analyze concept	Discuss literature + qual.	New conceptualization and fundamental propositions	New; cognitive, emotional, behavioral
(Dhanesh, 2017); customer	Analyze concept	Discuss literature	Need for broader definition; importance of certain (new) aspects	New; affective, cognitive, behavioral
(Graffigna and Gambetti, 2015); consumer brand	Analyze concept	Qual.	Identify concepts and process as experienced by customers	New (process); cognitive, affective, behavioral
(Hollebeek, 2011); customer brand	Analyze concept + new conceptualization	Discuss literature + qual.	New definition and key themes (immersion, passion and activation)	New; cognitive, emotional, behavioral
(Hollebeek et al., 2014); consumer brand	Evaluate concept + develop and validate scale	Qual. + quant.	Confirm concept; antecedents and consequences; scale validation	Existing; cognitive processing, affection, activation
(Kulta and Karjaluoto, 2016); mobile customer	Analyze concept in specific area	Systematic review	Two different conceptualizations (behavioral activity or holistic)	New; behavior, cognition, emotion
(Mittler et al., 2013); consumer	Apply to specific area + propose framework	Systematic review + case	Conceptual framework to classify engagement programs	New; activation, engaged behaviors
(Solem and Pedersen, 2016); Customer brand	Analyze concept + develop and test scale	Discuss literature + quant.	Components confirmed; antecedents and consequences	New; physical, emotional, cognitive
(Tan and Apisit-Isariyah, 2018); brand community	Analyze concept + develop model	Discuss literature + qual.	Typology and model with characteristics, antecedents and consequences	Unclear; cognitive, affective, behavioral, agentic/emphatic, para-social
(Yoshida et al., 2014); fan	Analyze concept in specific area + validate new scale	Quant.	Components confirmed; antecedents and consequences	New; management cooperation, individual participation, performance tolerance

When looking at the components, the same components (cognition, affect, and behavior) are found in eight studies, seemingly contradicting the need for a new definition in every study. When looking at these definitions (Appendix 1), it seems that many definitions convey a similar meaning (engagement as a multidimensional construct) but vary in what the different components entail, especially when applying the broad definition to a specific area. Interestingly, two studies explicitly see engagement more as a process than a state where the different components have a dynamic interplay that is more meaningful than the components in isolation (Bowden, 2014; Graffigna and Gambetti, 2015).

#### Health Engagement

Table 3 shows the 11 studies classified within the *health engagement* category. Within this category, there are numerous subtypes of which some (e.g., patient engagement, *n* = 3) seem to be broader than others (e.g., engagement in persons with dementia, *n* = 2; engagement in genetic testing, *n* = 1). The main purpose of seven studies is to analyze the concept of engagement, but there is also attention toward discussing and testing measures of engagement (*n* = 5). Most studies discuss or review literature (*n* = 8), and some use empirical data (qualitative, *n* = 3 and quantitative, *n* = 2) to gain more insight into the concept. There is an equal number of studies that formulate a new definition as studies that use an existing definition (*n* = 5), showing the breadth of the health engagement category.

**Table 3:** Characteristics and findings of health engagement studies.

**Study and subtype**	**Purpose**	**Method**	**Main findings**	**Definition and components**
(Bright et al., 2015); patient	Analyze concept	Systematic review	Conceptualization of engagement as a process and state	New (process and state); collaboration, contribution, active participation, emotional investment
(Cohen-Mansfield et al., 2009); persons with dementia	Analyze concept + new theoretical framework + test measure	Discuss literature + Quant.	Most important dimensions of engagement found	Unclear; refusal, attention, time, attitude, manipulating, holding
(Cohen-Mansfield et al., 2017); persons with dementia	Analyze concept + new theoretical framework + test measure	Discuss literature + Quant	Good psychometric properties of scale	Existing; attendance, attitude, active participation, asleep, group size, positive and negative interactions among group members
(Graffigna and Barello, 2018); patient	Discuss Patient Health Engagement (PHE) model and scale	Discuss literature	Process model seems valuable	Existing (process)
(Higgins et al., 2017); patient	Analyze and define concept	Systematic review	Four defining attributes	New (process); personalization, access, commitment, therapeutic alliance
(Macgowan, 2006); group	Discuss Group Engagement Measure (GEM)	Discuss literature	Multidimensional construct; good psychometric properties	Existing; attendance, individual participation, relating to worker and other members, contracting, working on own and other group members' problems
(McAllister, 2002); genetic testing	Explain behavior around predictive genetic testing (PGT)	Qual.	Engagement can explain variations in approaches and reactions to PGT	New; cognitive, individual participation
(Norris et al., 2017); stakeholder	Stakeholder perspective of concept	Qual.	Three main themes/attributes	New; individual participation, connecting around a purpose, meaningful interaction and dialog
(Pullmann et al., 2013); treatment	Analyze concept	Qual.	New definition	New; conduct, attitudes, relationships, empowerment, social context
(Staudt, 2007); treatment	Analyze concept and consequences	Discuss literature	More insight in the behavioral and attitudinal aspects	Existing; behavioral, attitudinal
(Yasui et al., 2017); mental health services	Analyze the role of culture in concept and measures	Systematic review	Limitations of current tools for minorities + new culturally infused model	Existing (process)

This breadth is further illustrated in the components used to describe engagement, as these vary widely. A behavioral component is seen most (*n* = 8), with participation used most frequently (*n* = 5). Five studies include more than one behavioral component. Next to the behavioral component, attitude is mentioned most often (*n* = 4). Lastly, there are four studies that see engagement as a process of which two do not identify any components.

#### Societal Engagement

Table 4 shows the characteristics of the ten studies classified as *societal engagement*. Within societal engagement, multiple subtypes are identified. These vary in whose engagement they define and measure, that is, the engagement of citizens (*n* = 6) (Nicotera et al., 2010; Kemp, 2015; Nguyen et al., 2016; Arvanitidis, 2017; Cortés-Cediel et al., 2018; Pontes et al., 2018) in, for example, their community, politics, or art; the engagement of organizations with citizens (*n* = 3) (Taylor and Kent, 2014; Sallnow and Paul, 2015; Eder et al., 2018), for example, engagement of research organizations with citizens, or of an end-of-life care service with the community surrounding it; or the engagement of interest groups in policy (*n* = 1) (Halpin and Fraussen, 2017). Four studies' main aim is to analyze the concept, but an equal number of studies apply the concept to a new area or seek a stakeholder perspective. Almost all studies discuss literature to achieve their aims (*n* = 8), but four use quantitative data and one includes qualitative data. Interestingly, only one study used an existing definition, whereas seven studies developed a new definition, often based on earlier definitions. Three studies mainly see engagement as a process, but all but one study identify multiple components of engagement.

**Table 4:** Characteristics and findings of societal engagement studies.

**Study and subtype**	**Purpose**	**Method**	**Main findings**	**Definition and components**
(Arvanitidis, 2017); civic	Analyze concept and antecedents	Discuss literature + quant.	Antecedents found	Existing; civic activities, electoral activities, political voice
(Cortés-Cediel et al., 2018); citizen	Present a process model	Discuss literature	Life cycle model different phases of engagement	New (process); intrinsic to the subject, intrinsic to the system, subjects' extrinsic motivations
(Eder et al., 2018); community	Stakeholder perspective of concept	Quant.	Different definitions used, but similar indicators and measures	None chosen (process)
(Halpin and Fraussen, 2017); policy	Analyze concept	Discuss literature	Identified forms of engagement	Unclear; involvement, access, prominence
(Kemp, 2015); arts	Analyze concept + develop measure	Discuss literature + quant.	Scale validated; antecedents and consequences	New; affective, cognitive, behavioral, social, connection
(Nguyen et al., 2016); community crowdsourcing	Propose behavioral perspective on definition and measurement	Discuss literature	Illustrate utility of the Participant Engagement Index	New; Activity, intensity, diversity, recency
(Nicotera et al., 2010); civic	Develop and validate scale for new target group (preadolescents)	Quant.	Components confirmed and specified	New; foundation for civic ethics, community connection
(Pontes et al., 2018); political	Stakeholder perspective of concept	Discuss literature + qual.	New definition; example actions and behaviors	New; cognitive, emotional
(Sallnow and Paul, 2015); community	Apply concept to specific topic; present model and definition	Discuss literature	New model with types of engagement	New (process): inform, consult, co-production, collaborate, empower
(Taylor and Kent, 2014); dialogic	Analyze concept within dialogue theory	Discuss literature	New definition and conceptualization, fitting in dialogue theory	New; individual participation, relational purpose, advice, contribute

Looking at these components, it is difficult to find commonalities, which might be due to the different target groups of these forms of engagement. However, there seem to be quite a few components of engagement that relate to behavior (e.g., civic or online activities). Furthermore, not all components seem to really reflect what engagement is but are motivations to be engaged (e.g., intrinsic motivations to the subject and system), goals of engagement (e.g., to inform or consult), or preconditions to be able to be engaged (e.g., access and prominence).

#### Work Engagement

Table 5 shows the nine studies categorized as *work engagement*. Most studies discuss or review literature to analyze the concept and related issues, whereas one uses qualitative data. Within this field, we found three different concepts: work engagement, personal engagement, and employee engagement (in one paper further specified as organization engagement). It seems that in literature, these concepts are sometimes used interchangeably, but a firm need is expressed to use the appropriate concept in the appropriate context (Shuck et al., 2017; Gupta and Sharma, 2018).

**Table 5:** Characteristics and findings of work engagement studies.

**Study and subtype**	**Purpose**	**Method**	**Main findings**	**Definition and components**
(Bakker et al., 2008); work	Introduce concept of work engagement	Discuss literature	Components, antecedents and consequences	Existing: vigor, dedication, absorption
(Bargagliotti, 2012); work	Apply concept to new context	Systematic review	Antecedents and consequences	Existing: vigor, dedication, absorption
(Green et al., 2017); work	Analysis of the concept + new theory	Discuss literature	Components; framework with antecedents	New: energy, positive experience, behavior
(Gupta and Sharma, 2018); employee	Analysis of the concept + measures	Systematic review	Differences in concepts, predictive validity and utility between scales	None chosen: cognitive, affective, physical strength, social, behavioral
(Kahn, 1990); personal	Theory construction	Qual.	Defined the concept, its components, antecedents and consequences	New: physical, cognitive, and emotional connection
(Schaufeli and Salanova, 2011); work	Analyze specific issues of concept	Discuss literature	More conceptual clarity on specific issues	Existing: vigor, dedication, absorption
(Shuck et al., 2017); employee	Analyze concept and compare to existing frameworks	Systematic review	New framework, need to differ between forms of engagement	New: cognitive, emotional, and behavioral energy
(Sonnentag, 2017); work	Analyze concept from task-level perspective	Discuss literature	Engagement varies between tasks and is not the opposite of from burnout	Existing: vigor, dedication, absorption
(Welch, 2011); employee, organization	Analyze concept and communication as antecedent	Discuss literature	New model with antecedents	New: emotional, cognitive, physical

For work engagement, there is one dominant (operational) definition, based on the Utrecht Work Engagement Scale (UWES) (Bakker et al., 2008). This definition, with the concepts vigor, dedication, and absorption, is used in four of five studies on work engagement. The components have also been categorized as energy, behavior, or physical engagement (vigor); emotion (dedication); and cognition (absorption). The discussion within this concept is mainly on specific issues (e.g., whether work engagement is the opposite of burnout) and less on the definition of the concept itself. For employee engagement, different new definitions have been proposed. The used components mirror the components of work engagement (e.g., cognitive, emotional, and behavioral energy), but other components have also been identified (e.g., social behavior). Interestingly, all studies within this category see engagement as a state and none as a process.

#### Digital Engagement

Table 6 shows the characteristics of the eight papers categorized as digital engagement. Four papers focus on (general) user engagement, three specifically on digital gaming (of which two on learning games), and one on engagement to digital behavior change interventions. Seven papers discussed or reviewed literature to analyze the concept, whereas three papers (also) used empirical data for this goal. In five studies, a new definition of engagement was constructed, based on the results of the study. Only one paper used an existing definition from literature, one study reviewed definitions but did not choose or construct one itself, and in one study, it was unclear what the chosen definition was based upon. In two papers, engagement is (also) seen as a process.

**Table 6:** Characteristics and findings of user engagement studies.

**Study and subtype**	**Purpose**	**Method**	**Main findings**	**Definition and components**
(Bouvier et al., 2014); digital gaming	Analyze concept + characterize behavior	Discuss literature	Define related concepts + characterize engaged behaviors	New; emotion, affect, thought
(Doherty and Doherty, 2018); user	Analyze concept + antecedents, consequences and measurements	Systematic review	Interpretation and measurement of engagement should be based on the context	None chosen
(Drejing et al., 2015); user	Propose definition and framework	Discuss literature	New framework and definition + propose way to measure it	New; behavior
(Kappelman and McLean, 1994); user	Analyze concept	Discuss literature	Identify four types of engagement	Unclear; participation, involvement
(Ke et al., 2016); game-based learning	Analyze concept + its development	Discuss literature + Qual.	New definition	New (process); affect, cognition, content, gameplay relevance
(O'Brien and Toms, 2008); user	Analyze concept + propose definition and operationalization	Discuss literature + qual.	New process and attributes of engagement	New (process and state); interest, motivation, affect, attention, challenge, feedback, aesthetics and sensory appeal, awareness, novelty, perceived control, perceived time, interactivity
(Perski et al., 2017); digital behavior change interventions	Analyze concept + develop framework	Systematic review	New definition + antecedents and consequences	New; behavior, attention, interest, affect
(Phillips et al., 2014); game-based learning	Expand definition and measurement of concept	Qual. + quant.	Highlight the importance of components	Existing; behavior, cognition, affect

Seven papers consider engagement to exist of one or more components. Affect or emotion is mentioned in five papers, as well as cognition or related concepts (thought, interest, and attention). Behavior or participation is mentioned in four papers. Looking at the various definitions (Appendix 1), engagement seems to be a much-debated concept in this field, and there seems to be no accepted definition. The two most recent studies both strive to tackle this issue using a systematic review but arriving a two seemingly different conclusions: whereas Perski et al. created a new definition for their specific target area (digital behavior change interventions) (Perski et al., 2017), Doherty and Doherty stated that the field needs to move away from identifying one definition of engagement and that it is more important to select the most useful interpretation and measurement of engagement, based on the context (Doherty and Doherty, 2018).

#### Transdisciplinary Engagement

One study was classified as covering *transdisciplinary engagement* as it employs a systematic review to integrate literature on employee, consumer, and patient engagement to find overlap between the concepts (c.f. Table 7). The study concludes that there are similarities between the fields of engagement, for example, in that the concept is seen as consisting of multiple components of which emotional, cognitive, and behavioral are most apparent.

**Table 7:** Characteristics and findings of transdisciplinary engagement study.

**Study and subtype**	**Purpose**	**Method**	**Main findings**	**Definition and components**
(Graffigna, 2017); trans-disciplinary	Analyze the concept in different fields	Systematic review	Five propositions that show overlap between employee, consumer and patient engagement	Unclear; emotional, cognitive, behavioral

## Discussion

The purpose of this systematic scoping review was to investigate in what domains the concept of engagement features and what constitutes engagement in these different domains and to determine whether there are any common components that seem to be important. With the 69 papers we identified on the conceptualization of engagement, we have identified seven different domains of engagement: student, customer, health, societal, work, digital, and transdisciplinary engagement. The results showed that engagement is a maturing concept that stretches across disciplinary boundaries. However, it seems as though some disciplines (e.g., organizational psychology) have a more crystalized view of such than others (e.g., within political sciences and sociology). Despite the level of maturity within a given discipline, our results showed that engagement is predominantly seen as a multidimensional construct, which is composed of a cognitive, behavioral, and affective component.

### Engagement Across Domains

A first observation is that engagement is viewed as an important concept across different domains but is also much disputed as seen by the many papers that analyze this concept. This resonates with the discussion on engagement in the field of eHealth technologies, or digital interventions (e.g., Yardley et al., 2016; Perski et al., 2017; Short et al., 2018). It seems that some fields are more mature regarding their conceptualization and theorizing on engagement than others. In particular, the field of work engagement seems to have a widely accepted definition (Bakker et al., 2008), but even in that area, there are numerous discussions surrounding the concept, for example, what the antipode is, what the attributing conditions are, and what the relationship is with employee/personal engagement. One of the aspects that seemed to have matured the domain of work engagement is the use of a commonly accepted measurement scale (UWES; Schaufeli et al., 2006), which is something that is not found in other fields. Other fields, for example, customer and societal engagement, seem to be somewhat less mature, in that they are in the phase of defining engagement as evidenced by the many new definitions that have been proposed in literature.

In all domains, engagement is mainly seen as a state of being engaged with something, but almost all domains also refer to engagement as a process. This process of getting engaged, staying engaged, disengaging, and re-engaging is sometimes viewed as more important that defining what the state of engagement really is (e.g., Bowden, 2014; Graffigna and Gambetti, 2015). However, it seems that by not separating the process of engagement from the state of engagement, antecedents for engagement can be confused for being part of engagement itself. Examples are digital engagement, where aesthetics have been proposed to be part of engagement (O'Brien and Toms, 2008), but recognized as antecedent or predictor of engagement in other studies (Short et al., 2018), and also societal engagement where motivations, goals, and preconditions are sometimes viewed as being part of engagement (Sallnow and Paul, 2015; Halpin and Fraussen, 2017; Cortés-Cediel et al., 2018).

### Engagement as a Multidimensional Construct

The results further show that across different domains, engagement is predominantly seen as a multidimensional construct comprising behavioral, cognitive, and affective components. There seems to be consensus on the combination of these three components in the domains of student and work engagement. Moreover, in customer and digital engagement, this combination is also seen quite often, although there does not seem to be consensus on the simultaneous manifestation or combination of these components (yet). In contrast, conceptualizations within health engagement seem to place more emphasis on the behavioral component (e.g., participation), but there is an ongoing discussion that engagement should be more than just doing something (Bright et al., 2015; Graffigna and Barello, 2018), which is similar to the discussion seen in engagement with eHealth technologies (Perski et al., 2017; Short et al., 2018). Similarly, societal engagement places emphasis on the behavioral component of engagement (e.g., various activities; Arvanitidis, 2017); however, recent discussions within the literature seem to point to engagement being more than just involvement or participation in societal activities (Pontes et al., 2018).

Although there is congruence between different domains as to the presence of behavioral, cognitive, and emotional components of engagement, the content of such differs significantly. Even within mature domains, such as work engagement, there are still debates into the psychological conditions or activities that categorize each of these components. For example, Kahn (1990) indicated that the physical/behavioral component of engagement refers to the extent toward which an individual can express himself or herself physically in a work role, whereas Bakker et al. (2008) argued that vigor (the physical/behavioral component in his model) refers to physical energy derived from work. The content of such differs even more between domains such as student engagement vs. work engagement. It is therefore understandable that different conceptualizations and definitions of engagement exist within and between different domains. Various studies aim to clarify what is meant by the behavioral, emotional, and cognitive components of engagement within a given context. This is done by either (a) constructing an operational or context-specific definition or approach of engagement or (b) employing a general meta-level model for engagement. The former results in a proliferation of definitions of engagement, which is difficult to keep track of or to maintain, and the latter results in a meta-level construct that ignores the context-specific challenges, experiences, or attributing factors. Arguably, an approach that lies between both options holds most merit for conceptualizing engagement in a new domain as eHealth interventions.

### Implications for Engagement With eHealth Technologies

Interest in understanding engagement within different disciplines has been increasing during the past three decades. Despite agreement between domains as to its importance, it is clear that theoretical discussions as to its conceptualization is ongoing within the literature. Controlling for context and discipline, it would seem as though engagement is predominantly seen as a *state* of being involved in or occupied with an object, activity, or artifact, which usually results in a positive outcome. This is part of the larger *process* of engagement.

Second, to go beyond the meta-construct of engagement, which ignores the context-specific challenges, experiences, or attributing factors and at the same time avoid a proliferation of definitions of engagement, which is difficult to keep track of or to maintain, it seems that the field needs a clear, tailored, and domain specific definition of the construct, which captures the associated emotional, behavioral, and cognitive components present within the given context. Questions that may need to be answered to arrive at this domain specific definition are, for example, whether behavioral engagement includes just the amount of usage or whether it should also include the quality of usage, for example, using as intended (Sieverink et al., 2017); whether interest and attention are the relevant cognitive aspects (Perski et al., 2017) or should other concepts be considered as, for example, involvement (Kelders et al., 2018) or “macro-engagement” (Yardley et al., 2016); and whether only positive emotions such as enjoyment should be seen as affective engagement or might negative affect also play a role, for example, when experiencing through eHealth technology that you have not reached your health-related goals (Triberti et al., 2018). In particular, when investigating the role of affect, a complicating factor is whether we should distinguish between experiences that are brought about by the content of the intervention and those that are triggered by the design of the intervention, for example, the sensory pleasure that people might derive from interacting with the intervention or the meaning they attribute to a particular feature in the design and that influences their affect. This is an area that has not received much attention but may give us more insight in the interplay between the design and content of interventions.

Ultimately, this may lead to a context-specific definition of engagement on a lower abstraction level, with an appropriate measurement method. However, it stands to reason that the more detailed the specification of the components will become, the more it will be aimed at one specific form of eHealth technologies. As the eHealth domain is very broad (e.g., encompassing interventions with or without care professionals; various devices and technologies; and various contexts), it is still an open question whether it is possible to gain a sufficiently detailed specification of the behaviors, cognitions, and affect that constitute engagement that is still broad enough to encompass the breath of the eHealth domain or whether there should be multiple specifications (and definitions) for subdomains. Therefore, it remains important for each individual study to be clear about what they mean by engagement.

Having a more commonly accepted understanding of the different components of engagement for eHealth technologies could allow a more structured investigation of how different technologies and forms of eHealth impact engagement; for example, in what way does blended care, or new forms of technology such as wearables, interactive devices, and virtual and augmented reality impact (the different components of) engagement? Also, new questions may then be addressed; for example, do people have different styles of being engaged; are some more inclined to be behaviorally engaged and others more affectively engaged (Kelders and Kip, 2019)? This might shed more light on why certain strategies, as, for example, gamification, work for some but not for others.

## Strengths and Limitations

This review set out to give an overview of how engagement is conceptualized in different fields. This is both the major strength and limitation of the review. By encompassing many different fields, a comprehensive overview of conceptualizations is given, which can inspire researchers in the field of eHealth technologies and beyond to use the concept of engagement in a substantiated way. However, owing to the large scope of the review, we needed to limit the search to papers that indicate in the title their focus on the concept of engagement. This may have caused us to miss papers that provide interesting insights on engagement but whose main focus was other than that. However, we feel that we have overcome this limitation by including many overview and review papers that do take these primary studies into account.

## Data Availability Statement

All datasets generated for this study are included in the article/Supplementary Material.

## Author Contributions

SK, LZ, and GL contributed to the conception and design of the study. SK performed the search, analysis, and wrote the first draft of the manuscript. LZ and GL contributed to the search and analysis. LZ and GL wrote sections of the manuscript. All authors contributed to manuscript revision, read and approved the submitted version.

## Conflict of Interest

The authors declare that the research was conducted in the absence of any commercial or financial relationships that could be construed as a potential conflict of interest.
