# Supplementary material for: The Concept and Components of Engagement in Different Domains Applied to eHealth: A Systematic Scoping Review
Source: Front Psychol. 2020 May 27;11:926. doi: 10.3389/fpsyg.2020.00926 (PMC7266981; doi:10.3389/fpsyg.2020.00926)
Supplement: Supplementary file 1 [file Table_1.DOCX]

# Appendix 1 - Definitions of engagement used in the included articles

Student engagement (n = 18)

| **Study** | **Used definition** |
| --- | --- |
| Appleton 2008 | No specific definition used |
| Barkaoui 2015 | Unclear, but models of student engagement tend to emphasize the psychological and behavioral dimensions of the concept, focusing on student characteristics and school factors, but ignoring demographic and community factors. |
| Bernard 2015 | a dynamic process marked by a positive behavioral, cognitive, and affective state exhibited in the pursuit of deep learning. |
| Burch 2015 | consists of the separate constructs of emotional engagement, physical engagement, cognitive engagement in class, and cognitive engagement out of class. |
| Ciric 2016 | 1) encompasses students’ sense of belonging and connectedness to their school, teachers and peers, 2) encompasses students’ sense of agency, self-efficacy and orientation to achieve within their classrooms and in their broader extracurricular endeavors, 3) encompasses students’ involvement, effort, levels of concentration and interest in subjects and learning in general, 4) the extent to which learning is enjoyed for its own sake or seen as something that must be endured to receive a reward or avoid sanction, 5) a variable state of being that is influenced by a range of internal and external factors including the perceived value or relevance of the learning and the presence of opportunities for students to experience appropriately-pitched challenge and success in their learning, 6) malleable by the actions of teachers. |
| Fredricks 2004 | Researchers describe behavioral, emotional, and cognitive engagement and recommend studying engagement as a multifaceted construct. |
| Harris 2008 | Unclear |
| Harris 2011 | Unclear |
| Hollingshead 2018 | In general, scholars agree that academic (i.e., behavioral) engagement refers to behaviors displayed by students in the context of a classroom and in relation to academic tasks. Cognitive engagement is described in terms of investment in attempting “more complex ideas and mastering difficult skills”. Emotional engagement is also often defined in relation to students’ social interactions and motivation to participate in learning tasks. |
| Jimerson 2003 | a multifaceted construct that includes affective, behavioral, and cognitive dimensions that are contextually bound |
| Lawson 2013 | a dynamic system of social and psychological constructs as well as a synergist. We present it as the conceptual glue that connects student agency (including students' prior knowledge, experience, and interest at school, home, and in the community) and its ecological influences (peers, family, and community) to the organizational structures and cultures of school |
| Liem 2012 | motivation is defined as individuals’ energy and drive to learn, work effectively, and achieve to their potential, and engagement as the behaviours aligned with this energy. |
| Montenegro 2017 | agentic engagement: the observable classroom event in which the learner constructively contributes to his/her learning and the instruction he/she receives |
| Reeve 2013 | agentic engagement: intentional, purposive student initiated action to render the learning environment to become more motivationally supportive. |
| Schuetz 2008 | a state of interest, mindfulness, cognitive effort, and deep processing of new information that partially mediates the gap between what learners can do and what they actually do. |
| Skinner 2009 | includes behavioral and emotional participation in the classroom and requires a conceptualization of its opposite, which we term disaffection |
| Unrau 2014 | defined in terms of the actions (both observable and unobservable) associated with a person’s reading activities. This definition of reading engagement maintains a multidimensional perspective, with affective, behavioral, and cognitive components. |
| Wang 2017 | behavioral, emotional, cognitive, and social indicators. engagement and disengagement in school emerged as two related, but potentially distinct constructs |

Customer engagement (n = 11)

| **Study** | **Used definition** |
| --- | --- |
| Abdul-Ghani 2018 | It’s a motivational construct that prompts ongoing participation in the consumption of a product. It’s a multidimensional construct comprised of cognitive and affective reactions to the consumption experience; identification with, and motivation to repeat, the consumption experience. |
| Bowden 2009 | The process of engagement traces the temporal development of loyalty by mapping the relationships between the constructs of calculative commitment, affective commitment, involvement, and trust as customers progress from being new to a service brand to becoming repeat purchasers of a specific service brand. |
| Brodie 2011 | a psychological state and multidimensional concept subject to a context- and/or stakeholder-specific expression of relevant cognitive, emotional and/or behavioral dimensions. |
| Dhanesh 2017 | an affective, cognitive, and behavioral state wherein publics and organizations who share mutual interests in salient topics interact along continua that range from passive to active and from control to collaboration, and is aimed at goal attainment, adjustment, and adaptation for both publics and organizations. |
| Graffigna 2015 | a dynamic process that evolves in three progressive relational phases: friendship, intimacy and symbiosis. |
| Hollebeek 2011 | the level of a customer’s cognitive, emotional and behavioral investment in specific brand interactions, which are displayed by applying particular levels of brand-related concentration, positive affect and energy (time/effort) in specific brand interactions |
| Hollebeek 2014 | a consumer's positively valanced brand-related cognitive, emotional and behavioral activity during or related to focal consumer/brand interactions |
| Kulta 2016 | a multidimensional concept including behavioral, emotional, and cognitive dimensions. |
| Mittler 2013 | the performance of specific behaviors (“engaged behaviors”) and/or an individual’s capacity and motivation to perform these behaviors (“activation”). |
| Solem 2016 | a customer’s motivational and positive state of mind, characterized by physical, emotional and cognitive investments in brand relationships. |
| Tan 2018 | Unclear |
| Yoshida 2014 | a sport consumer’s extra-role behaviors in non-transactional exchanges to benefit his or her favorite sport team, the team’s management, and other fans; a multidimensional, behavioral construct composed of management cooperation, prosocial behavior, and performance tolerance. |

Health engagement (n = 11)

| **Study** | **Used definition** |
| --- | --- |
| Bright 2015 | a co-constructed process and state. It incorporates a process of gradually connecting with each other and/or a therapeutic program, which enables the individual to become an active, committed and invested collaborator in healthcare. |
| Cohen-Mansfield 2009 | the act of being occupied or involved with an external stimulus |
| Cohen-Mansfield 2017 | the act of being occupied or involved with an external stimulus |
| Graffigna 2018 | a developmental process involving a recovered patient’s capacity to plan realistic life projects (ie, meaningful life aims that the patient might generate unless living with the disease condition) and to be goal oriented – even if living with a disease. The process of involves four developmental phases, namely, blackout, arousal, adhesion, and eudaimonic project. |
| Higgins 2017 | patient engagement is a developmental process involving a recovered patient’s capacity to plan realistic life projects (ie, meaningful life aims that the patient might generate unless living with the disease condition) and to be goal oriented – even if living with a disease. The process of patient engagement as described by the PHE model involves four developmental phases, namely, blackout, arousal, adhesion, and eudaimonic project. |
| Macgowan 2006 | Consists of seven domains: attendance, contributing, relating to group worker, relating with group members, contracting with the group service, working on own problems, working on other group members' problems |
| McAllister 2002 | a newly constructed concept reflecting the degree of cognitive and emotional involvement with cancer risk in individuals from these families, and models the psychosocial process of engaging with cancer risk |
| Norris 2017 | an active and committed decision-making about a meaningful problem through respectful interactions and dialog where everyone’s voice is considered |
| Pullmann 2013 | CARES definition: Conduct, Attitudes, Relationships, Empowerment, and Social Context |
| Staudt 2007 | No specific definition chosen, but most of the definitions of engagement (and related terms) include behaviors (session attendance, homework completion) and either explicitly reference, or allude to, an attitudinal component. |
| Yasui 2017 | No specific definition chosen, but mainly seen as a process |

Societal engagement (n = 10)

| **Study** | **Used definition** |
| --- | --- |
| Arvanitidis 2017 | How “an active citizen participates in the life of a community in order to improve conditions for others or to help shape the community’s future” (Adler & Goggin, 2005, p. 241); including wide ranging activities undertaken alone or in concert with others designed to identify and address issues of public concern. |
| Cortés-Cediel 2018 | a cycle with various phases, where one can perform particular mechanisms to stimulate the citizens’ participation at different levels. |
| Eder 2018 | None chosen, but this definition was used most frequently by the stakeholders: the process of working collaboratively with and through groups of people affiliated by geographic proximity, special interest, or similar situations to address issues affecting the well-being of those people. . . . It often involves partnerships and coalitions that help mobilize resources and influence systems, change relationships among partners, and serve as catalysts for changing policies, programs, and practices. |
| Halpin 2017 | Unclear |
| Kemp 2015 | the feelings, cognitions, behavioral, social, and connective/relational responses evoked by artistic experiences |
| Nguyen 2016 | the magnitude, temporal intensity, diversity, and recency of tangible effort online users voluntarily devote to what is requested in a community crowdsourcing initiative |
| Nicotera 2010 | A sense of connection to and responsibility for others with whom one shares a geographic locality (Atkins & Hart, 2003) combined with collaboration and participation (Flanagan, 2003; Lerner, Fisher & Weinberg, 2000; Lerner et al.2003; Pittman et al., 2003) that foster connection and responsibility across multicultural differences (Flanagan 2003; Lerner et al., 2003; Torney-Purta, 2002). |
| Pontes 2018 | having interest in, paying attention to, having knowledge or opinions about, being conscious of, proactive about and constantly informed about politics |
| Sallnow 2014 | a process which enables communities and services to work together to understand, build capacity and address issues to improve their experience of end-of-life and bereavement and their related well being. It exists on a spectrum of engagement that extends from informing through to empowering, depending on a range of factors such as the degree of participation from the local community and the intention of the work. Community engagement activities by end-of-life care services go beyond working in the community to working with the community to improve its experience of end-of-life care. |
| Taylor 2014 | a two-way, relational, give-and-take between organizations and stakeholders/publics with the intended goal of (a) improving understanding among interactants; (b) making decisions that benefit all parties involved, not simply the organization; and (c) fostering a fully functioning society (Heath, 2006), where decisions are made based on informed participative interactions that involve stakeholders. |

Work engagement (n= 9)

| **Study** | **Used definition** |
| --- | --- |
| Bakker 2008 | A specific, well defined, and properly operationalized psychological state that is open to empirical research and practical application: a positive, fulfilling, work-related state of mind that is characterized by vigour, dedication, and absorption |
| Bargagliotti 2012 | Work engagement is a positive, fulfilling state of mind about work that is characterized by vigour, dedication and absorption. In nursing, work engagement is the dedicated, absorbing, vigorous nursing practice that emerges from settings of autonomy and trust and results in safer, cost effective patient outcomes. |
| Green Jr 2017 | First, we identified the key attribute of energy. Second, we identified that positive emotional experiences are the source of the energy so key to the sustenance of work engagement. Finally, we argued that work engagement has been systematically theorized or empirically demonstrated as a behavioral phenomenon—an experience leading to important positive organizational outcomes. |
| Gupta 2018 | None chosen |
| Kahn 1990 | the harnessing of organisation members' selves to their work roles; in engagement, people employ and express themselves physically, cognitively, and emotionally during role performances. |
| Schaufeli 2011 | Unclear, but “Given the operational definition of work engagement as implied by the UWES, we agree with the lead article that vigour and dedication constitute its core dimensions.” |
| Shuck 2017 | a positive, active, work-related psychological state operationalized by the maintenance, intensity, and direction of cognitive, emotional, and behavioral energy. To be sure that we have been as clear as possible, employee engagement is a unique framework. That is our ardent position. Conceptually speaking, it is not synonymous with anything else, nor is it empirically redundant |
| Sonnentag 2017 | the experience of vigor, dedication and absorption (Schaufeli & Bakker, 2004). A task-specific approach to work engagement emphasizes the understanding of engagement as an experience that happens in the process of working, on a day-to-day basis. |
| Welch 2011 | a dynamic, changeable psychological state which links employees to their organisations, manifest in organisation member role performances expressed physically, cognitively and emotionally, and influenced by organisation-level internal communication |

Digital engagement (n = 8)

| **Study** | **Used definition** |
| --- | --- |
| Bouvier 2014 | The willingness to have emotions, affect and thoughts directed towards and aroused by the mediated activity in order to achieve a specific objective which is categorized by environmental-, social-, self and action- engagement. |
| Doherty 2018 | No specific definition used |
| Dreijing 2015 | The magnitude of an intrinsically motivated behavior that is initiated by an organism to reach a specific goal. |
| Kappelman 1994 | The total set of user relationships toward information systems and their development, implementation and use. |
| Ke 2016 | An integrated and continuing process that advance from affective involvement, cognitive engagement, to content engagement, and a dynamic state that reflects the connection between players and their gaming environment. |
| O’Brien 2008 | A quality of user experience characterized by attributes of challenge, positive affect, endurability, aesthetic and sensory appeal, attention, feedback, variety/novelty, interactivity, and perceived user control. |
| Perski 2017 | (1) the extent (e.g. amount, frequency, duration, depth) of usage and (2) a subjective experience characterized by attention, interest and affect. |
| Philips 2014 | Being viewed along three axes: behavioral, cognitive, and affective. |

General engagement (n = 1)

| **Study** | **Used definition** |
| --- | --- |
| Graffigna 2017 | Unclear, but: the analysis cast light on the following areas of conceptual overlap among employee, consumer and patient engagement: (1) engagement is different from empowerment and activation; (2) engagement is a multi-componential psychological experience; (3) engagement is a self-transformative experience; (4) engagement develops within a relational context; and (5) engagement is a systemic phenomenon. |
